# Association of leptin levels and relative leptin deficiency with steatotic liver disease in the general population

**DOI:** 10.1007/s12020-025-04429-y

**Published:** 2025-09-16

**Authors:** Bernhard Wernly, Marianna Beghini, Georg Semmler, Vera Paar, Michael Lichtenauer, Franz Singhartinger, Andreas Völkerer, Mathias Ausserwinkler, Maria Flamm, Elmar Aigner, Thomas Scherer, Christian Datz

**Affiliations:** 1 Institute of General Practice, Family Medicine and Preventive Medicine, Paracelsus Medical University of Salzburg, Salzburg, 5020 Austria; 2 Department of Internal Medicine 1, Paracelsus Medical University, Salzburg, Austria; 3 Division of Endocrinology & Metabolism, Department of Medicine III, Medical University of Vienna, Vienna, Austria; 4 Division of Gastroenterology and Hepatology, Department of Medicine III, Medical University of Vienna, Vienna, Austria; 5 Department of Internal Medicine II, Salzburg State Hospital (SALK), Salzburg, Austria; 6 Department of Surgery, Paracelsus Medical University, Salzburg, Austria; 7 Hospital Oberndorf, Oberndorf, Austria

**Keywords:** Leptin, Steatotic liver disease, MASLD, Obesity, Metabolic syndrome

## Abstract

**Background:**

Leptin replacement therapy has shown promising results in treating metabolic steatohepatitis (MASH) patients with leptin levels below the 25th percentile for their sex and BMI category (“relative leptin deficiency”, RLD). However, the clinical utility of the RLD definition for identifying high-risk individuals for liver disease in general screening populations remains unclear.

**Methods:**

We analyzed the association between leptin levels and steatotic liver disease (SLD) in 636 participants from the screening population of the SAKKOPI registry (2007–2020) who underwent metabolic phenotyping, leptin measurement, and liver assessment including ultrasound, controlled attenuation parameter (CAP), and transient elastography (kPa). SLD was categorized as MASLD, MetALD, ALD, cryptogenic SLD, or no SLD. RLD was defined using NHANES III-based sex- and BMI-specific cutoffs. Leptin was analyzed categorically (RLD vs. non-RLD) and continuously, including leptin-to-BMI ratios (ln[leptin]/ln[BMI]) to assess leptin as a marker independent of body mass.

**Results:**

Each two-fold increase in leptin was independently associated with MASLD (adjusted RRR 1.58, 95% CI 1.20–2.09, *p* = 0.001) and MetALD (RRR 1.74, 95% CI 1.02–2.98, *p* = 0.043). Leptin-to-BMI ratio analysis confirmed this relationship, with the highest quintile showing 83.7 dB/m higher CAP values compared to the lowest quintile (*p* < 0.001). Individuals classified as having RLD (n = 112, 18%) demonstrated significantly better metabolic health and lower SLD prevalence (27% vs. 48%, p < 0.001). The leptin/BMI ratio showed specificity for steatosis parameters (CAP, fatty liver index) but no association with liver stiffness (kPa, p = 0.55) or APRI. However, FIB-4 scores were higher in the lowest leptin/BMI quintile (1.27 vs. 1.11, p = 0.011), an association that persisted after age and sex adjustment.

**Conclusions:**

In this screening cohort of the general population, NHANES III-derived RLD thresholds may not effectively identify high-risk individuals for SLD in this population, but rather appear to select metabolically healthier participants. While the persistent FIB-4 association deserves consideration, the absence of corresponding associations with direct fibrosis measurements suggests caution in interpretation. These findings indicate that current RLD definitions appear to have limited utility for risk stratification in this specific screening population, though their applicability in high-risk clinical settings requires further investigation.

## Introduction

Metabolic dysfunction-associated steatotic liver disease (MASLD), represents one of the most prevalent liver conditions worldwide, affecting approximately 30% of the global population [1]. The spectrum ranges from simple steatosis to steatohepatitis (MASH) with potential progression to cirrhosis and hepatocellular carcinoma. Despite this substantial disease burden, there are currently limited approved pharmacotherapies for NAFLD/MASLD, with lifestyle modifications remaining the primary therapeutic approach [2]. The identification of novel biomarkers and therapeutic targets is therefore an urgent clinical priority.

Leptin, an adipokine primarily secreted by adipose tissue, plays a central role in energy homeostasis and hepatic lipid metabolism [3, 4]. Recent studies have shown that leptin promotes hepatic lipid export, thereby protecting against hepatic steatosis [5]. Under physiological conditions, leptin reduces hepatic lipogenesis and promotes fatty acid oxidation, thereby protecting against hepatic steatosis [5]. In rare conditions of severe leptin deficiency, such as congenital or acquired lipodystrophy, patients develop severe hepatic steatosis and MASH [6]. Leptin replacement therapy in these patients leads to improvements in liver histology, with significant reductions in steatosis, inflammation, and hepatocellular injury [7].

Beyond these established roles in severe deficiency states, emerging evidence highlights the complex genetic and molecular mechanisms underlying leptin’s involvement in MASLD pathogenesis. Genetic factors, particularly variations in the leptin receptor (LepR) gene, significantly influence insulin sensitivity and lipid metabolism, contributing to MASLD susceptibility and progression [8]. Recent murine studies demonstrate that LepR mutations can directly impact MASLD development, underscoring the importance of intact leptin signaling pathways [8]. Additionally, mounting evidence suggests that leptin may play a role in hepatic fibrogenesis, with circulating leptin concentrations showing independent correlations with liver fibrosis severity in overweight and obese individuals [9]. These findings expand our understanding of leptin’s multifaceted role beyond simple steatosis regulation to encompass fibrotic progression in MASLD.

Recent clinical evidence has expanded the potential therapeutic window for leptin beyond severe deficiency states. Akinci et al. demonstrated that metreleptin therapy significantly improved NASH scores not only in patients with partial lipodystrophy but also in males with “relative leptin deficiency” (RLD) - defined as leptin levels below the 25th percentile for body mass index and sex based on US population data [10]. This finding suggests that the therapeutic utility of leptin may extend to more common forms of liver disease, challenging the traditional view that leptin resistance limits its clinical application in obesity-related comorbidities.

However, the concept of RLD and its clinical relevance in broader populations remains incompletely understood. The original RLD criteria were derived from a US population (NHANES III) and validated in selected NASH patients, raising questions about their generalizability to European populations and unselected screening cohorts [10]. Furthermore, the relationship between RLD and steatotic liver disease in real-world settings has not been systematically characterized. Understanding whether RLD represents a clinically meaningful subgroup could inform risk stratification strategies and identify patients who might benefit from leptin-based interventions.

We therefore conducted a comprehensive analysis in a large, representative Central European screening cohort to examine the association between leptin levels, RLD status, and steatotic liver disease. Our objectives were to: (1) validate the RLD criteria in a European population, (2) characterize the relationship between leptin levels and MASLD prevalence, and (3) assess the potential utility of leptin as a biomarker for hepatic steatosis risk stratification in real-world clinical practice. The distinction between pathological leptin deficiency and constitutively low but physiologically adequate leptin levels may be crucial for clinical application.

## Materials and methods

### Study population

This retrospective analysis utilized data from participants in the “Salzburg Colon Cancer Prevention Initiative” (SAKKOPI), a prospective registry study conducted at the General Hospital in Oberndorf, Austria, between January 2007 and March 2020 [11, 12]. The original cohort comprised 6,154 asymptomatic individuals from the general population who underwent colorectal cancer screening through either primary care physician referral or self-enrollment in an opportunistic screening program fully funded by health insurance. All participants provided written informed consent for the use of their data for scientific research purposes. For the present analysis, we included only participants with complete data for leptin levels, body mass index, transient elastography or steatosis assessment and sex, resulting in a final study population of 636 individuals.

### Clinical assessment and data collection

All participants underwent a standardized two-day clinical assessment. On the first day, comprehensive medical histories were obtained through structured interviews, vital signs were recorded using certified monitors, and physical examinations were performed by trained clinical staff. Height, weight, and waist and hip circumferences were measured by nursing staff, with body mass index automatically calculated using the medical software system (Patidok 2.0, Professional Clinical Software GmbH, Klagenfurt, Austria). Additional lifestyle and demographic information was collected through paper-based questionnaires covering dietary habits, physical activity, smoking status, alcohol consumption, and family medical history.

Laboratory analyses were performed using the hospital’s internal laboratory and included comprehensive metabolic panels, liver function tests, lipid profiles, glucose metabolism markers (including HbA1c and oral glucose tolerance testing), and inflammatory markers. Leptin levels were measured using standard enzyme-linked immunosorbent assay (ELISA) methodology. All participants underwent standardized abdominal ultrasound examination and transient elastography (FibroScan) to assess liver steatosis using the Controlled Attenuation Parameter (CAP) and liver stiffness measured in kilopascals (kPa).

### Definition of steatotic liver disease

Participants were classified into mutually exclusive categories based on clinical and laboratory findings. Metabolic dysfunction-associated steatotic liver disease (MASLD) was defined according to current consensus criteria, requiring evidence of hepatic steatosis with at least one cardiometabolic risk factor [13]. Metabolic dysfunction and alcohol-associated liver disease (MetALD) was diagnosed in participants meeting MASLD criteria who also consumed 140–350 g of alcohol per week for females or 210–420 g per week for males. Alcohol-associated liver disease (ALD) was defined as hepatic steatosis with alcohol consumption exceeding these thresholds. Cryptogenic steatotic liver disease was diagnosed when hepatic steatosis was present without identifiable metabolic or alcohol-related causes. Participants without evidence of hepatic steatosis were classified as having no steatotic liver disease.

### Leptin level classification

Leptin levels were measured using standard enzyme-linked immunosorbent assay (ELISA) methodology (R&D Systems, Minneapolis, MN, USA). Other laboratory analyses were performed using the hospital’s internal laboratory with certified assays.

Leptin levels were analyzed both as a continuous variable and using established categorical definitions. For continuous analyses, leptin values were natural log-transformed and scaled by dividing by the natural logarithm of 2, allowing interpretation of results as the effect per two-fold increase in leptin concentration. Participants were also classified by median leptin levels, with those above the population median (8.94 ng/mL) considered to have elevated leptin levels. The association between leptin and HbA1c was examined using linear regression with natural log-transformed leptin levels scaled by ln(2) to allow interpretation as effects per two-fold increase in leptin concentration.

Relative leptin deficiency (RLD) was defined using established criteria derived from the 25th percentile of leptin levels stratified by body mass index and sex from the United States National Health and Nutrition Examination Survey III (NHANES III) population [10]. Specifically, RLD was defined as leptin levels below the following thresholds: for women with BMI less than 25 kg/m², less than 5.0 ng/mL; BMI 25.0–27.4 kg/m², less than 12.0 ng/mL; BMI 27.5–29.9 kg/m², less than 14.0 ng/mL; BMI 30.0–34.9 kg/m², less than 18.0 ng/mL; and BMI 35 kg/m² or greater, less than 25.7 ng/mL. For men, the corresponding thresholds were: BMI less than 25 kg/m², less than 2.0 ng/mL; BMI 25.0–27.4 kg/m², less than 3.2 ng/mL; BMI 27.5–29.9 kg/m², less than 4.0 ng/mL; BMI 30.0–34.9 kg/m², less than 7.0 ng/mL; and BMI 35 kg/m² or greater, less than 10.9 ng/mL.

### Statistical analysis

Continuous variables were assessed for normality and reported as median with interquartile range or mean with standard deviation as appropriate. Categorical variables were presented as frequencies and percentages. Comparisons between groups were performed using the Mann-Whitney U test for non-parametric continuous variables, Student’s t-test for parametric continuous variables, and chi-square tests for categorical variables. The association between leptin levels and steatotic liver disease categories was examined using multinomial logistic regression, with no steatotic liver disease as the reference category. Results were reported as relative risk ratios (RRR) with 95% confidence intervals. Three progressive models were fitted: Model 1 included leptin levels alone; Model 2 additionally adjusted for individual metabolic syndrome components (triglycerides, HDL cholesterol, hypertension, glucose levels) and body mass index as continuous variable. Interaction analyses were performed to assess effect modification by age, sex, metabolic syndrome status, body mass index, and individual metabolic parameters. To assess leptin efficiency independent of body mass, leptin-to-BMI ratios were calculated as ln(leptin)/ln(BMI) and analyzed both as continuous variables and categorized into quintiles. Linear regression was used to examine associations between leptin/BMI ratios and CAP values, with pairwise comparisons between quintiles performed using Bonferroni correction for multiple testing. Age and sex adjusted analyses were performed for all liver parameters using linear regression, with results reported as adjusted coefficients and 95% confidence intervals. For participants with steatotic liver disease, linear regression analyses examined the relationship between leptin levels and quantitative measures of liver involvement, including CAP values for hepatic steatosis and liver stiffness measurements for fibrosis assessment. Separate analyses were conducted for each steatotic liver disease subtype. All statistical tests were two-tailed, and p-values less than 0.05 were considered statistically significant. Analyses were performed using StataNow version 19.5 (StataCorp LLC, College Station, TX, USA).

### Ethical approval

This study was conducted in accordance with the principles of the Declaration of Helsinki. The study protocol was approved by the local Ethics Committee for the province of Salzburg (approval no. 415-E/1262). All participants provided written informed consent for participation and the use of their data for scientific research purposes.

## Results

Of the 6,154 participants in the SAKKOPI cohort, 636 individuals had complete data for leptin levels, body mass index, and sex. Leptin levels showed a right-skewed distribution with a median of 8.94 ng/mL (IQR 4.88-17.00), characterized by marked positive skewness (2.12) and high kurtosis (10.18), consistent with typical adipokine distributions.

Participants were stratified by median leptin levels to examine baseline characteristics (Table 1). Those with leptin above the median were predominantly female (71% vs. 28%, *p* < 0.001) and had significantly higher adiposity measures, including BMI (28 vs. 25 kg/m², *p* < 0.001) and obesity prevalence (33% vs. 6%, *p* < 0.001). The high leptin group demonstrated substantially worse metabolic parameters across all domains, including glycemic control, lipid profiles, and blood pressure, resulting in higher metabolic syndrome prevalence (91% vs. 84%, *p* = 0.008). Inflammatory burden was also elevated, as evidenced by higher C-reactive protein levels.

Leptin demonstrated significant associations with several metabolic parameters (Table 1). Each two-fold increase in leptin was independently associated with higher HbA1c levels (β = 0.49, 95% CI 0.29–0.69, *p* < 0.001, R²=0.036). Leptin showed no significant associations with age (*p* = 0.39), triglyceride levels (*p* = 0.073), or elevated blood pressure (*p* = 0.16), but was inversely associated with alcohol consumption ≥ 2 drinks/day (*p* = 0.043)

**Table 1:** Baseline characteristics by leptin levels

Characteristic	Total	Leptin ≤ Median	>Median	*p*-value
	*N* = 636	*N* = 325	*N* = 311	
**Demographic Characteristics**				
Age (years)	57 (53–63)	57 (53–63)	57 (53–64)	0.39
Male sex	51% (324)	72% (235)	29% (89)	< 0.001
**Anthropometric Parameters**				
Body mass index (kg/m²)	26 (24–29)	25 (23–27)	28 (25–31)	< 0.001
BMI ≥ 30 kg/m²	19% (122)	6% (18)	33% (104)	< 0.001
Waist circumference (cm)	93 (85–102)	91 (82–98)	96 (86–106)	< 0.001
Elevated waist circumference*	66% (413)	47% (151)	86% (262)	< 0.001
**Metabolic Parameters**				
HbA1c (%)	5.4 (5.2–5.6)	5.3 (5.1–5.5)	5.5 (5.3–5.7)	< 0.001
Fasting glucose (mg/dL)	95 (88–102)	95 (88–101)	95 (89–105)	0.049
Diabetes status				
No diabetes	54% (345)	57% (186)	51% (159)	0.008
Prediabetes	35% (220)	35% (115)	34% (105)	
Diabetes	11% (71)	7% (24)	15% (47)	
Triglycerides ≥ 150 mg/dL†	29% (185)	26% (84)	33% (101)	0.073
Low HDL cholesterol‡	10% (63)	6% (19)	14% (44)	< 0.001
Systolic blood pressure (mmHg)	140 (126–150)	140 (125–150)	140 (128–150)	0.26
Diastolic blood pressure (mmHg)	80 (79–90)	80 (76–89)	80 (80–90)	0.036
Elevated blood pressure§	77% (492)	75% (244)	80% (248)	0.16
**Individual MetS Criteria**				
BMI criterion	75% (476)	61% (197)	90% (279)	< 0.001
Glucose criterion	65% (413)	61% (197)	69% (216)	0.020
Blood pressure criterion	75% (479)	74% (241)	77% (238)	0.49
Triglycerides criterion	29% (185)	26% (84)	32% (101)	0.066
HDL criterion	22% (138)	16% (51)	28% (87)	< 0.001
**Metabolic syndrome (≥ 3 criteria)**	87% (542)	84% (267)	91% (275)	0.008
**Lifestyle Factors**				
Smoking status				
Never smoker	53% (336)	56% (180)	51% (156)	0.29
Ex-smoker	31% (195)	28% (91)	34% (104)	
Active smoker	16% (98)	16% (52)	15% (46)	
Alcohol ≥ 2 drinks/day	4% (27)	6% (19)	3% (8)	0.043
**Inflammation Marker**				
C-reactive protein (mg/dL)	0.1 (0.1–0.3)	0.1 (0.1–0.2)	0.2 (0.1–0.4)	< 0.001

Data are presented as median (interquartile range) for continuous variables and as percentage (number) for categorical variables. The leptin median was 8.94 ng/mL. P-values were calculated using Mann-Whitney U test for continuous variables and chi-square test for categorical variables

MetS denotes metabolic syndrome, BMI body mass index, HDL high-density lipoprotein

*Elevated waist circumference was defined as > 102 cm in men and > 88 cm in women

†Triglycerides ≥ 150 mg/dL represents one component of metabolic syndrome criteria

‡Low HDL cholesterol was defined as < 40 mg/dL in men and < 50 mg/dL in women

§Elevated blood pressure was defined as systolic ≥ 130 mmHg, diastolic ≥ 85 mmHg, or use of antihypertensive medication

**Fig. 1:**
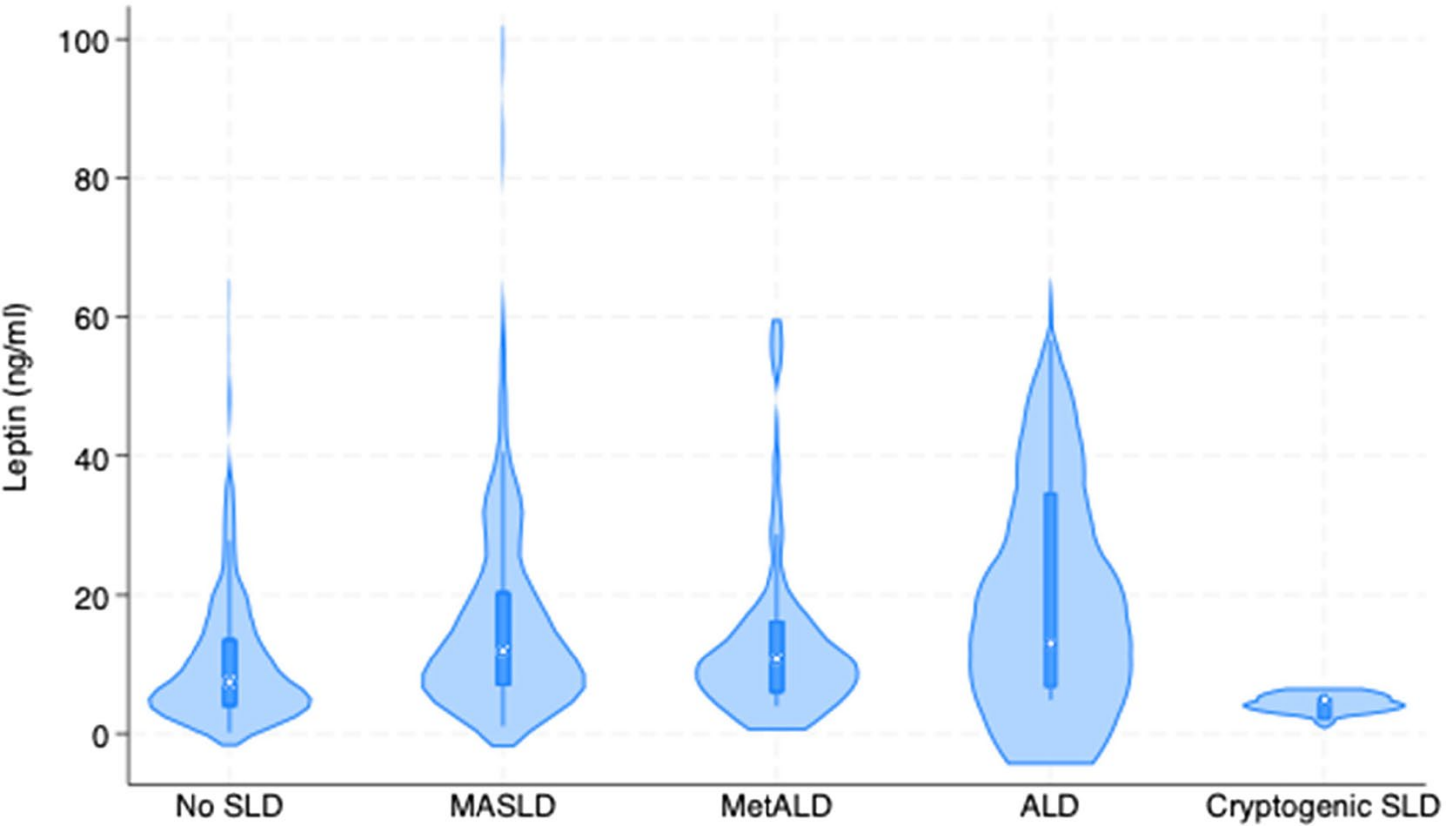
Distribution of serum leptin concentrations across steatotic liver disease (SLD) categories. Violin plots show the probability density distribution of leptin levels (ng/ml) for each SLD group. The width of each violin represents the frequency of observations at that leptin concentration. Central markers indicate median values. No SLD: median 7.4 ng/ml (*n* = 354); MASLD: median 11.9 ng/ml (*n* = 236); MetALD: median 10.7 ng/ml (*n* = 31); ALD: median 13.0 ng/ml (*n* = 12); Cryptogenic SLD: median 4.9 ng/ml (*n* = 3). Leptin concentrations showed progressive elevation from No SLD to ALD groups, with the highest variability observed in the ALD cohort. MASLD = Metabolic dysfunction-associated steatotic liver disease; MetALD = Metabolic and alcohol-related liver disease; ALD Alcohol-related liver disease

Hepatic involvement showed a clear gradient across leptin levels (Table 2). MASLD prevalence was significantly higher with elevated leptin (44% vs. 30%, *p* < 0.001), while quantitative steatosis measures confirmed this relationship with higher CAP values (270 vs. 241 dB/m, *p* < 0.001) and fatty liver index scores. Fig. 1 demonstrates the distribution of serum leptin concentrations across steatotic liver disease categories, showing progressive elevation from No SLD (median 7.4 ng/ml) to ALD (median 13.0 ng/ml), with the highest variability observed in the ALD cohort. Fibrosis markers showed an inverse pattern, with lower FIB-4 scores in the high leptin group. Total bilirubin levels were significantly lower in participants with higher leptin levels (0.6 vs. 0.8 mg/dL, *p* < 0.001)

**Table 2:** Liver-related parameters by leptin levels

	Tota	≤ Median	>Median	*p*-value
**Parameter**	*N* = 636	*N* = 325	*N* = 311	
**Liver Disease Classification**				
SLD type				
No SLD	56% (354)	63% (205)	48% (149)	< 0.001
MASLD	37% (236)	30% (98)	44% (138)	
MetALD	5% (31)	4% (14)	5% (17)	
ALD	2% (12)	2% (5)	2% (7)	
Cryptogenic SLD	0% (3)	1% (3)	0% (0)	
**Hepatic Steatosis**				
CAP (dB/m)	254 (217–303)	241 (211–287)	270 (227–321)	< 0.001
Elevated CAP	39% (244)	32% (104)	46% (140)	< 0.001
Fatty liver index	40 (16–70)	34 (13–57)	49 (20–78)	< 0.001
FLI categories				
FLI < 30	41% (252)	46% (146)	35% (106)	< 0.001
FLI 30–60	28% (172)	33% (106)	22% (66)	
FLI ≥ 60	32% (196)	21% (66)	43% (130)	
**Liver Fibrosis**				
Liver stiffness (kPa)	4.5 (3.6–5.6)	4.5 (3.6–5.5)	4.6 (3.6–5.7)	0.35
Elevated liver stiffness	5% (32)	4% (13)	6% (19)	0.22
FIB-4 score	1.05 (0.85–1.30)	1.10 (0.88–1.42)	1.01 (0.83–1.24)	0.001
FIB-4 categories				
FIB-4 < 1.30	74% (472)	67% (219)	81% (253)	< 0.001
FIB-4 1.30–2.66	24% (155)	31% (102)	17% (53)	
FIB-4 ≥ 2.67	1% (9)	1% (4)	2% (5)	
**Liver Parameter**				
AST (U/L)	20 (17–24)	21 (18–24)	19 (16–24)	0.001
ALT (U/L)	21 (16–28)	21 (16–28)	20 (15–28)	0.57
Total bilirubin (mg/dL)	0.7 (0.5–0.9)	0.8 (0.6-1.0)	0.6 (0.5–0.8)	< 0.001

Data are presented as median (interquartile range) for continuous variables and as percentage (number) for categorical variables. The leptin median was 8.94 ng/mL. P-values were calculated using Mann-Whitney U test for continuous variables and chi-square test for categorical variables. Elevated CAP was defined as ≥ 248 dB/m. Elevated liver stiffness was defined as ≥ 7.0 kPa

SLD denotes steatotic liver disease, MASLD metabolic dysfunction-associated steatotic liver disease, MetALD metabolic dysfunction and alcohol-associated liver disease, ALD alcohol-associated liver disease, CAP controlled attenuation parameter, FLI fatty liver index, FIB-4 fibrosis-4 index, AST aspartate aminotransferase, ALT alanine aminotransferase

Individuals classified as having relative leptin deficiency demonstrated significantly different metabolic profiles compared to those without deficiency (Table 3). RLD participants had lower BMI (25 vs. 26 kg/m², *p* < 0.001), reduced waist circumference (86 vs. 94 cm, *p* < 0.001), and better glycemic control with lower HbA1c levels (5.3 vs. 5.4%, *p* = 0.002) and fasting glucose (91 vs. 96 mg/dL, *p* < 0.001). They also showed more favorable lipid profiles with lower triglyceride prevalence (20% vs. 31%, *p* = 0.016) and reduced inflammatory burden as evidenced by lower C-reactive protein levels (*p* < 0.001)

**Table 3:** Baseline characteristics by leptin deficiency status

	No Deficiency	Deficiency	*p*-value
**Characteristic**	*N* = 524	*N* = 112	
**Demographic Characteristics**			
Age (years)	57 (53–63)	58 (53–64)	0.44
Male sex	51% (268)	50% (56)	0.83
**Anthropometric Parameters**			
Body mass index (kg/m²)	26 (24–29)	25 (22–27)	< 0.001
BMI ≥ 30 kg/m²	20% (107)	13% (15)	0.086
Waist circumference (cm)	94 (87–103)	86 (77–94)	< 0.001
Elevated waist circumference*	72% (370)	39% (43)	< 0.001
**Metabolic Parameters**			
HbA1c (%)	5.4 (5.2–5.6)	5.3 (5.2–5.5)	0.002
Fasting glucose (mg/dL)	96 (89–104)	91 (86–97)	< 0.001
Diabetes status			
No diabetes	52% (274)	63% (71)	0.095
Prediabetes	36% (188)	29% (32)	
Diabetes	12% (62)	8% (9)	
Triglycerides ≥ 150 mg/dL†	31% (163)	20% (22)	0.016
Low HDL cholesterol‡	10% (55)	7% (8)	0.28
Systolic blood pressure (mmHg)	140 (128–150)	135 (125–150)	0.18
Diastolic blood pressure (mmHg)	80 (80–90)	80 (75–86)	0.004
Elevated blood pressure§	79% (416)	68% (76)	0.008
**Individual MetS Criteria**			
BMI criterion	79% (413)	56% (63)	< 0.001
Glucose criterion	67% (352)	54% (61)	0.010
Blood pressure criterion	77% (404)	67% (75)	0.024
Triglycerides criterion	31% (163)	20% (22)	0.015
HDL criterion	23% (121)	15% (17)	0.065
**Metabolic syndrome (≥ 3 criteria)**	90% (462)	74% (80)	< 0.001
**Lifestyle Factors**			
Smoking status			
Never smoker	52% (271)	59% (65)	0.25
Ex-smoker	32% (168)	24% (27)	
Active smoker	15% (79)	17% (19)	
Alcohol ≥ 2 drinks/day	5% (23)	4% (4)	0.65
**Inflammation Marker**			
C-reactive protein (mg/dL)	0.2 (0.1–0.3)	0.1 (0.0-0.2)	< 0.001

Data are presented as median (interquartile range) for continuous variables and as percentage (number) for categorical variables. Relative leptin deficiency was defined using NHANES III-derived sex- and BMI-specific 25th percentile cutoffs. P-values were calculated using Mann-Whitney U test for continuous variables and chi-square test for categorical variables

MetS denotes metabolic syndrome, BMI body mass index, HDL high-density lipoprotein, NHANES National Health and Nutrition Examination Survey

*Elevated waist circumference was defined as > 102 cm in men and > 88 cm in women

†Triglycerides ≥ 150 mg/dL represents one component of metabolic syndrome criteria

‡Low HDL cholesterol was defined as < 40 mg/dL in men and < 50 mg/dL in women

§Elevated blood pressure was defined as systolic ≥ 130 mmHg, diastolic ≥ 85 mmHg, or use of antihypertensive medication

Consequently, RLD individuals had significantly lower metabolic syndrome prevalence (74% vs. 90%, *p* < 0.001) and substantially reduced MASLD prevalence (21% vs. 40%, *p* < 0.001; Table 4). RLD individuals showed higher bilirubin levels (0.8 vs. 0.7 mg/dL, *p* < 0.001), though values remained within normal range.

**Table 4:** Liver-related parameters by leptin deficiency status

	No Deficiency	Deficiency	*p*-value
**Parameter**	*N* = 524	*N* = 112	
**Liver Disease Classification**			
SLD type			
No SLD	52% (272)	73% (82)	< 0.001
MASLD	40% (212)	21% (24)	
MetALD	5% (27)	4% (4)	
ALD	2% (12)	0% (0)	
Cryptogenic SLD	0% (1)	2% (2)	
**Hepatic Steatosis**			
CAP (dB/m)	264 (223–307)	224 (198–254)	< 0.001
Elevated CAP	43% (223)	19% (21)	< 0.001
Fatty liver index	44 (20–73)	18 (8–49)	< 0.001
FLI categories			
FLI < 30	35% (180)	67% (72)	< 0.001
FLI 30–60	30% (152)	19% (20)	
FLI ≥ 60	35% (180)	15% (16)	
**Liver Fibrosis**			
Liver stiffness (kPa)	4.5 (3.6–5.6)	4.5 (3.6–5.6)	0.94
Elevated liver stiffness	5% (28)	4% (4)	0.43
FIB-4 score	1.03 (0.84–1.28)	1.16 (0.92–1.43)	0.007
FIB-4 categories			
FIB-4 < 1.30	76% (397)	67% (75)	0.15
FIB-4 1.30–2.66	23% (120)	31% (35)	
FIB-4 ≥ 2.67	1% (7)	2% (2)	
**Liver Parameter**			
AST (U/L)	20 (17–24)	21 (17–24)	0.17
ALT (U/L)	21 (16–29)	18 (14–24)	< 0.001
Total bilirubin (mg/dL)	0.7 (0.5–0.9)	0.8 (0.6-1.0)	< 0.001

Data are presented as median (interquartile range) for continuous variables and as percentage (number) for categorical variables. Relative leptin deficiency was defined using NHANES III-derived sex- and BMI-specific 25th percentile cutoffs. P-values were calculated using Mann-Whitney U test for continuous variables and chi-square test for categorical variables. Elevated CAP was defined as ≥ 248 dB/m. Elevated liver stiffness was defined as ≥ 7.0 kPa

SLD denotes steatotic liver disease, MASLD metabolic dysfunction-associated steatotic liver disease, MetALD metabolic dysfunction and alcohol-associated liver disease, ALD alcohol-associated liver disease, CAP controlled attenuation parameter, FLI fatty liver index, FIB-4 fibrosis-4 index, AST aspartate aminotransferase, ALT alanine aminotransferase, NHANES National Health and Nutrition Examination Survey

### Leptin and steatotic liver disease risk

In univariate multinomial logistic regression, each two-fold increase in leptin was associated with significantly increased risk across multiple SLD categories: MASLD (RRR 1.55, 95% CI 1.34–1.79, *p* < 0.001), MetALD (RRR 1.49, 95% CI 1.10–2.02, *p* = 0.011), and ALD (RRR 1.96, 95% CI 1.19–3.23, *p* = 0.009). Cryptogenic SLD showed a non-significant inverse association (RRR 0.59, 95% CI 0.27–1.28, *p* = 0.181)

In fully adjusted models controlling for age, sex, and ATP III metabolic syndrome components (triglycerides ≥ 150 mg/dL, HDL cholesterol < 40 mg/dL in men or < 50 mg/dL in women, blood pressure ≥ 130/85 mmHg or treatment, glucose ≥ 100 mg/dL) plus BMI, leptin remained independently associated with MASLD (RRR 1.58, 95% CI 1.20–2.09, *p* = 0.001) and MetALD (RRR 1.74, 95% CI 1.02–2.98, *p* = 0.043). The association with ALD was attenuated and no longer significant (RRR 1.42, 95% CI 0.60–3.37, *p* = 0.423). Cryptogenic SLD continued to show no association (RRR 0.68, 95% CI 0.18–2.52, *p* = 0.561)

### Leptin interaction effects

Interaction analyses revealed several important effect modifiers of the leptin-SLD relationship. Sex significantly influenced leptin associations, with females demonstrating stronger leptin effects across all SLD categories. In sex-stratified models, each leptin doubling in females was associated with a 3.18-fold MASLD increase (95% CI 2.26–4.47, *p* < 0.001), while the male interaction term was non-significant (RRR 0.91, 95% CI 0.58–1.42, *p* = 0.687), indicating comparable effects after accounting for baseline sex differences. Similar patterns emerged for MetALD with strong female associations (RRR 2.45, 95% CI 1.27–4.71, *p* = 0.007) and non-significant male modification (RRR 1.08, 95% CI 0.47–2.48, *p* = 0.855), and for ALD with the strongest female effect (RRR 4.72, 95% CI 1.71–13.06, *p* = 0.003) and attenuated male interaction (RRR 0.54, 95% CI 0.14–2.03, *p* = 0.363)

Metabolic syndrome status also modified leptin effects on MASLD risk. Participants without metabolic syndrome showed weaker leptin-MASLD associations (RRR 0.81, 95% CI 0.45–1.46, *p* = 0.485), while those with metabolic syndrome demonstrated enhanced susceptibility with a significant interaction effect (RRR 1.88, 95% CI 1.03–3.43, *p* = 0.041). MetALD and ALD showed similar directional but non-significant patterns

BMI significantly modified leptin effects specifically for MetALD risk. Higher BMI was associated with increased baseline MetALD risk (RRR 2.08 per unit BMI, 95% CI 1.37–3.17, *p* = 0.001), while the leptin-BMI interaction suggested diminishing leptin effects at higher BMI levels (interaction RRR 0.89, 95% CI 0.80–0.99, *p* = 0.027). MASLD and ALD showed non-significant BMI interactions. Glucose levels significantly enhanced leptin effects on MetALD risk, with the interaction term indicating that hyperglycemia potentiated leptin’s association with MetALD (RRR 1.98, 95% CI 1.04–3.79, *p* = 0.038), while effects on other SLD categories remained unmodified

Age, triglyceride levels, HDL cholesterol, and hypertension did not significantly modify leptin associations with any SLD category, with all interaction terms yielding non-significant p-values across disease states

### Quantitative steatosis analysis

Among participants with specific SLD categories, leptin demonstrated differential associations with hepatic fat quantification. In MASLD patients (*n* = 232), each leptin doubling was associated with 11.5 dB/m higher CAP values in univariate analysis (95% CI 5.2–17.8, *p* < 0.001), increasing to 23.5 dB/m after full adjustment (95% CI 16.0–31.0, *p* < 0.001), explaining 19% of steatosis variance in the adjusted model

In MetALD patients (*n* = 30), univariate analysis showed a non-significant 9.8 dB/m increase per leptin doubling (95% CI -6.3 to 25.8, *p* = 0.223). After adjustment, the association remained non-significant (13.4 dB/m, 95% CI -6.3 to 33.1, *p* = 0.174), though the fully adjusted model explained 34% of steatosis variance

ALD patients (*n* = 12) showed no crude association with CAP (-6.5 dB/m, 95% CI -40.1 to 27.2, *p* = 0.677), but a significant positive association emerged after adjustment (41.5 dB/m, 95% CI 3.8 to 79.1, *p* = 0.035), with the adjusted model explaining 70% of steatosis variance

### Leptin-to-BMI ratio analysis

To comprehensively explore the relationship between relative leptin levels and hepatic parameters independent of population-derived cut-offs, we analyzed leptin levels normalized by BMI as a continuous measure (ln[Leptin]/ln[BMI]). This approach aimed to capture leptin efficiency while avoiding the limitations of arbitrary threshold definitions

The leptin/BMI ratio demonstrated a strong linear association with hepatic steatosis in continuous analysis (β = 48.8 dB/m per unit increase in CAP, 95% CI 30.0-67.6, *p* < 0.001, R²=0.040). Quintile analysis revealed consistent dose-response relationships across multiple liver parameters (Table 5).

**Table 5:** Liver parameters across leptin-to-BMI ratio quintiles

Parameter	Q1 (*n* = 128)	Q2 (*n* = 127)	Q3 (*n* = 127)	Q4 (*n* = 127)	Q5 (*n* = 127)	*p*-trend	Q5 vs. Q1 (95% CI)	Adj *p*-value*
**Leptin/BMI ratio†**	0.29 (0.16)	0.53 (0.04)	0.68 (0.04)	0.82 (0.04)	1.01 (0.08)	< 0.001	-	-
**Steatosis Parameters**								
CAP (dB/m)	236.5 (56.0)	250.3 (64.7)	263.5 (61.2)	270.8 (70.2)	270.5 (58.2)	< 0.001	+ 83.7 (60.9-106.5)	< 0.001
FLI	32.3 (22.5)	40.9 (27.8)	45.3 (30.2)	46.3 (31.8)	53.7 (29.6)	< 0.001	+ 54.4 (45.7–63.2)	< 0.001
**Fibrosis Parameters**								
kPa	4.87 (2.10)	4.63 (1.35)	4.92 (1.95)	5.00 (1.83)	4.79 (1.91)	0.55	+ 0.55 (0.03–1.07)	0.368
FIB-4	1.27 (0.52)	1.15 (0.44)	1.16 (0.43)	1.07 (0.43)	1.11 (0.55)	0.011	-0.16 (-0.28 to -0.03)	0.118
APRI	0.259 (0.11)	0.251 (0.09)	0.239 (0.11)	0.225 (0.09)	0.220 (0.10)	0.29	+ 0.019 (-0.03-0.07)	0.408
NFS	-1.74 (1.17)	-1.57 (1.08)	-1.47 (1.16)	-1.65 (1.18)	-1.26 (1.20)	0.015	+ 0.96 (0.55–1.37)	< 0.001

Data are presented as mean (standard deviation). The leptin-to-BMI ratio was calculated as ln(leptin)/ln(BMI). P-trend values were calculated using linear regression. Age and sex adjusted analyses were performed using linear regression with Bonferroni correction for multiple comparisons

CAP denotes controlled attenuation parameter, FLI fatty liver index, FIB-4 fibrosis-4 index, APRI AST-to-platelet ratio index, NFS NAFLD fibrosis score, BMI body mass index

*P-values are adjusted for age and sex with Bonferroni correction for multiple comparisons

†Leptin/BMI ratio values represent quintile means

For steatosis parameters, we observed clear progressive associations. CAP values increased systematically from the lowest to highest leptin/BMI quintiles (236.5 to 270.5 dB/m, *p* < 0.001 for trend), with significant differences emerging from the third quintile onward compared to the reference group (Q3 vs. Q1: *p* = 0.007; Q4 vs. Q1: *p* < 0.001; Q5 vs. Q1: *p* < 0.001, all Bonferroni-adjusted). Age and sex adjustment strengthened these associations substantially, with the highest quintile showing 83.7 dB/m higher CAP values compared to the lowest quintile (95% CI 60.9-106.5, *p* < 0.001), and the explained variance increasing dramatically from R²=0.043 to R²=0.240. Similarly, the Fatty Liver Index demonstrated robust associations across quintiles, with the adjusted effect size of 54.4 points difference between extreme quintiles (95% CI 45.7–63.2, *p* < 0.001)

In contrast, structural fibrosis parameters showed divergent patterns. Liver stiffness measurements (kPa) demonstrated no consistent association across leptin/BMI quintiles in either unadjusted (F = 0.76, *p* = 0.55) or adjusted analyses (highest vs. lowest quintile: +0.55 kPa, 95% CI 0.03–1.07, *p* = 0.368 after Bonferroni correction). This specificity for steatosis rather than structural fibrosis was consistent with our primary leptin analyses

The FIB-4 score presented a unexpected pattern, with lower scores (indicating better liver health) observed in higher leptin/BMI quintiles. This inverse association was statistically significant in crude analysis (*p* = 0.011 for trend) and remained notable after age and sex adjustment, though the effect was attenuated (Q5 vs. Q1: -0.16, 95% CI -0.28 to -0.03, *p* = 0.118 after Bonferroni correction). This pattern was not observed with other fibrosis markers: APRI showed no significant association across quintiles (*p* = 0.29), while the NAFLD Fibrosis Score showed a positive association with higher leptin/BMI ratios after adjustment (*p* < 0.001)

### Liver fibrosis analysis

Leptin showed no consistent association with liver fibrosis markers (kPa measurements) across SLD categories. In MASLD patients, leptin doubling was not associated with kPa in univariate (0.02 kPa, *p* = 0.860) or adjusted models (0.29 kPa, *p* = 0.058). MetALD patients showed a significant positive association in univariate analysis (0.77 kPa, *p* = 0.045) that strengthened after adjustment (1.29 kPa, *p* = 0.019). ALD patients demonstrated no significant associations in either univariate (-0.27 kPa, *p* = 0.371) or adjusted models (-0.21 kPa, *p* = 0.530). These findings confirm leptin’s specific relationship with hepatic fat accumulation rather than fibrogenesis

### Leptin deficiency and SLD association

**Fig. 2:**
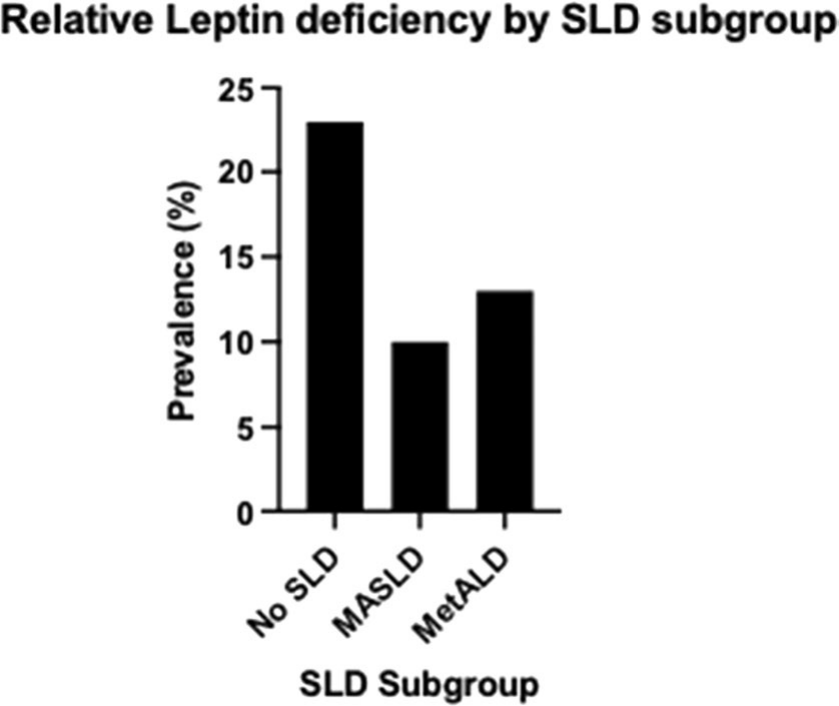
Prevalence of relative leptin deficiency across steatotic liver disease categories. Bar chart shows the percentage of participants meeting NHANES III-derived leptin deficiency criteria within each SLD subgroup. Leptin deficiency was most prevalent in participants without SLD (23.4%) and progressively decreased in MASLD (10.6%) and MetALD (13.8%)

Using NHANES III-derived criteria, 112 participants (18%) met relative leptin deficiency definitions. In univariate analysis, leptin deficiency was inversely associated with MASLD (RRR 0.38, 95% CI 0.23–0.61, *p* < 0.001), with non-significant effects on MetALD (RRR 0.49, 95% CI 0.17–1.45, *p* = 0.197) and negligible effects on ALD and Cryptogenic SLD. After full adjustment for ATP III metabolic syndrome components, age, sex, and BMI, leptin deficiency remained inversely associated with MASLD (RRR 0.48, 95% CI 0.27–0.86, *p* = 0.014), indicating that individuals classified as having constitutively low leptin levels showed reduced hepatic steatosis independent of overall metabolic health. Fig. 2 demonstrates the inverse relationship between leptin deficiency and SLD severity, with deficiency prevalence highest in No SLD participants (23.4%), intermediate in MASLD (10.6%) and MetALD (13.8%), and absent in ALD and Cryptogenic SLD groups

### Leptin deficiency interaction effects

Interaction analyses for leptin deficiency revealed age as a potential effect modifier for MetALD risk, with stronger inverse associations observed in older individuals (*p* = 0.074 for interaction), though this did not reach conventional statistical significance. Sex did not significantly modify leptin deficiency effects, with inverse associations with MASLD remaining consistent in females (RRR 0.28, 95% CI 0.12–0.69, *p* = 0.006) and a non-significant male interaction term (RRR 1.42, 95% CI 0.48–4.22, *p* = 0.528). BMI showed a trend toward modifying leptin deficiency associations, with enhanced inverse effects at lower BMI levels for MASLD (interaction RRR 0.89, 95% CI 0.77–1.03, *p* = 0.123), though confidence intervals included unity. Metabolic syndrome status, triglycerides, HDL cholesterol, hypertension, and glucose levels did not significantly modify leptin deficiency effects across any SLD category.

### Leptin deficiency and liver fibrosis

The relationship between leptin deficiency and liver fibrosis was examined across SLD categories. In MASLD patients (*n* = 234), leptin deficiency showed no significant association with liver stiffness in either univariate (-0.27 kPa, 95% CI -1.21 to 0.68, *p* = 0.578) or adjusted models (-0.32 kPa, 95% CI -1.28 to 0.64, *p* = 0.506). Similarly, in MetALD patients (*n* = 30), leptin deficiency was not significantly associated with fibrosis markers in univariate (-1.18 kPa, 95% CI -3.73 to 1.37, *p* = 0.352) or adjusted analyses (-1.40 kPa, 95% CI -4.50 to 1.70, *p* = 0.362). Among ALD patients (*n* = 12), leptin deficiency could not be evaluated due to absence of deficient individuals in this subgroup. These findings indicate that leptin deficiency status does not significantly influence liver fibrosis development across steatotic liver disease categories.

## Discussion

In this large, unselected screening cohort from Central Europe, we observed a robust, dose-dependent association between circulating leptin levels and steatotic liver disease. Each two-fold increase in leptin was independently associated with a 58% higher risk of MASLD, even after adjustment for metabolic risk factors and body mass index. Quantitative ultrasound-based steatosis measures mirrored these associations, with leptin explaining nearly one-fifth of CAP variance among MASLD participants. However, individuals classified as having relative leptin deficiency (RLD) by NHANES III-based criteria showed a substantially lower prevalence of MASLD and more favorable metabolic profiles—raising questions about the generalizability of RLD thresholds across populations.

The strong positive association between leptin and MASLD is consistent with the biological role of leptin as an adiposity-linked hormone [4, 14, 15]. Rather than conferring protection, elevated leptin likely reflects adipose tissue expansion in combination with leptin resistance, which in turn is linked to inflammation and metabolic dysfunction. Our data support this interpretation: higher leptin concentrations were associated with worse metabolic profiles, even after accounting for BMI and individual metabolic syndrome components.

Individuals classified as having RLD by NHANES III-based criteria showed a substantially lower prevalence of MASLD and more favorable metabolic profiles; therefore, although RLD patients with MASH benefit from leptin substitution, RLD thresholds did not appear to identify patients at high risk for SLD in our screening population. Our findings suggest that NHANES III-derived thresholds may not identify true leptin deficiency in this screening population [10]. Instead, individuals with leptin levels below these thresholds appear to represent a metabolically healthy subgroup with constitutively low but physiologically adequate leptin concentrations. This contrasts with the clinical NASH populations studied by Akinci et al., where low leptin may reflect pathological insufficiency in the context of severe metabolic dysfunction, which was ameliorated by leptin therapy. Several factors may account for this divergence [10]. First, our cohort differs substantially from Akinci’s clinical population: participants in our study were older, predominantly Caucasian, and enrolled through voluntary screening rather than hepatology referral. Second, RLD thresholds derived from NHANES III may not be transferable to European populations, where genetic, dietary, and environmental determinants of leptin levels differ. Application of these US-based cut-offs may inadvertently identify individuals with constitutively low—but physiologically normal—leptin concentrations, rather than those with true functional deficiency.

The utility of leptin as a continuous biomarker is further supported by our analysis of leptin-to-BMI ratios. This approach, which avoids arbitrary cut-offs while preserving the biological relationship between leptin and adiposity, demonstrated a clear dose-response relationship with hepatic steatosis. The plateau effect observed at higher leptin/BMI ratios provides additional evidence for leptin resistance in individuals with metabolic dysfunction, while the lowest ratio quintile—representing efficient leptin signaling relative to body mass—was associated with the least hepatic fat accumulation. In this regard, leptin appears to be a continuous biological marker for SLD.

However, these findings in a screening population should be interpreted within the broader context of leptin biology. Established evidence from lipodystrophy syndromes, “lipodystrophy-like” phenotypes and genome-wide association studies consistently demonstrates that true leptin deficiency states are associated with increased metabolic dysfunction and hepatic steatosis because in this context leptin represents a biomarker for reduced functional adipose tissue mass causing excess calories to be stored in ectopic sites which drives lipotoxicity and insulin resistance [16, 17]. The apparent “protective” effect of low relative leptin levels in our cohort likely reflects the absence of pathological leptin deficiency in this population, rather than contradicting fundamental leptin physiology. Our findings suggest that NHANES III-derived thresholds may identify individuals with constitutively low but physiologically adequate leptin levels, rather than those with clinically meaningful leptin insufficiency. This distinction between physiological variation and pathological deficiency may be crucial for identifying patients who would benefit from leptin-based interventions.

While our leptin-to-BMI ratio analysis confirms leptin’s utility as a continuous biomarker for hepatic steatosis, the clinical application of relative leptin deficiency definitions in screening populations remains complex. Our findings suggest that NHANES III-derived RLD thresholds are not effective for identifying steatosis risk groups in unselected populations, where they appear to capture metabolically healthy individuals rather than those with pathological leptin insufficiency.

However, the robust association between low leptin/BMI ratios and elevated FIB-4 scores deserves careful consideration. This finding aligns with established evidence from lipodystrophy syndromes and suggests that leptin deficiency may have prognostic implications for liver health that extend beyond simple steatosis. The fact that this association persisted after age and sex adjustment indicates it may represent a genuine biological signal rather than a statistical artifact. The specificity of this finding to FIB-4—while liver stiffness measurements (kPa) and APRI showed no consistent associations—raises questions about the underlying mechanism. These findings align with recent evidence demonstrating independent associations between circulating leptin levels and hepatic fibrosis in individuals with BMI ≥ 25 kg/m², suggesting that leptin’s role in liver pathophysiology extends beyond steatosis regulation to encompass fibrogenic processes [9]. The genetic underpinnings of this relationship are increasingly understood, with leptin receptor gene variants influencing not only insulin sensitivity and lipid metabolism but also MASLD progression through complex molecular pathways [8]. While our cross-sectional design precludes causal inference, the persistent association between low leptin/BMI ratios and elevated FIB-4 scores may reflect early dysregulation of leptin-mediated hepatoprotective mechanisms that warrant longitudinal investigation. The observed differences in bilirubin levels across leptin groups, while statistically significant, remained within normal physiological ranges and may reflect subtle variations in hepatic metabolism rather than clinically meaningful hepatic dysfunction, warranting cautious interpretation given our cross-sectional study design.

These findings highlight a fundamental limitation in applying population-based percentile thresholds to define hormonal deficiency. Low leptin in healthy individuals likely reflects efficient energy homeostasis and preserved adipose tissue function, rather than inadequate leptin signaling and reduced functional adipose tissue mass [18]. The therapeutic benefit of leptin replacement in clinical cohorts may thus be restricted to contexts where leptin production is truly insufficient relative to metabolic demand - a condition that may be rare in screening populations.

It is also conceivable that our findings reflect the inherent selection effects of screening cohorts. Individuals who participate in preventive health programs tend to be more health-literate and metabolically healthier than the general population. In this context, low leptin may indicate a leaner phenotype with preserved metabolic flexibility rather than subclinical leptin insufficiency [19]. Such selection dynamics could attenuate or even invert the expected relationship between RLD and hepatic steatosis observed in clinical cohorts with biopsy-proven NASH.

Despite this apparent paradox, our findings reinforce the potential utility of leptin as a continuous biomarker for hepatic steatosis risk. The observed associations were independent of BMI and metabolic syndrome, highlighting leptin’s potential role in refining MASLD risk stratification. However, our results also caution against dichotomous interpretation of leptin levels based on population-derived percentiles. The therapeutic implications of RLD—demonstrated in highly selected clinical cohorts—may not translate to broader screening populations, where leptin physiology remains within homeostatic bounds.

Our study is limited by its cross-sectional design, precluding causal inference. The generalizability of our findings is also constrained by the characteristics of our screening cohort. Longitudinal studies incorporating diverse populations are needed to clarify whether leptin trajectories predict hepatic disease progression and to determine whether population-specific reference ranges improve the identification of clinically meaningful leptin deficiency. Moreover, future research should explore whether leptin-based risk models can complement existing MASLD screening algorithms in real-world settings.

Taken together, our findings highlight a clear association between higher leptin concentrations and MASLD risk, while raising questions about the utility of current RLD definitions in similar European screening populations. The contrast with interventional data from clinical NASH cohorts underscores the importance of population context when applying biomarker thresholds. The absence of harmful effects in individuals with low leptin levels reflects the fact that NHANES III thresholds identify healthy individuals with constitutively low leptin rather than those with pathological deficiency—underscoring the need for recalibrated biomarker frameworks tailored to the setting in which they are applied.

## Data Availability

No datasets were generated or analysed during the current study.
